# Natural Rubber Composites Filled with Crop Residues as an Alternative to Vulcanizates with Common Fillers

**DOI:** 10.3390/polym11060972

**Published:** 2019-06-03

**Authors:** Marcin Masłowski, Justyna Miedzianowska, Krzysztof Strzelec

**Affiliations:** Institute of Polymer & Dye Technology, Lodz University of Technology, Stefanowskiego 12/16, 90-924 Lodz, Poland; justyna.miedzianowska@edu.p.lodz.pl (J.M.); krzysztof.strzelec@p.lodz.pl (K.S.)

**Keywords:** cereal straw, elastomer composites, natural rubber, carbon black, silica, chalk, talc, agriculture waste

## Abstract

This present study is focused on exploring the possibility of using agricultural waste in the form of cereal straw as an active filler of biocomposites. The effect of lignocellulosic filler addition on the multifunctional properties of natural rubber composites was investigated. The results were compared with the properties of vulcanizates containing commonly used reinforcements in elastomer technology (carbon black, silica, chalk, talc). Rubber mixtures filled with straw showed the highest torque increase during rheometric measurements, which indirectly indicated a high degree of crosslinking and hardness of composites. It was found that the effect of straw addition on vulcanization time of elastomer blends was comparable with the results obtained for other conventional fillers. Moreover, the results confirmed that novel composites based on natural rubber filled with crop residues were attractive materials owing to their capacity for the formation of “structure” in combination with a good impact on reinforcement. Vulcanizates with the addition of straw showed the best barrier properties and resistance to thermo-oxidative aging from all tested samples. Furthermore, straw-based composites demonstrated that cereal straw waste could be used as an alternative, biodegradable and eco-friendly reinforcement of natural rubber composites.

## 1. Introduction

Conventional rubber fillers are inorganic or organic substances added to the elastomer to improve its mechanical, dielectric, thermal, chemical, or processing properties and to reduce production costs by increasing the weight and volume of the product. The fillers’ function is to give the rubber mixtures the appropriate processing properties and the functional properties of the vulcanizates [1]. The most frequently used fillers include chalk, hydrocarbon fillers such as carbon black, graphite, carbon fiber, and also silicates like talc, kaolin, mica and many more [2,3]. The effect of filler addition on the elastomeric composition is determined by the type of surface and its energy status, presence of functional groups and their construction, agglomeration and aggregation capacity, the ability to create a structure, and its own spatial network [4]. Moreover, one of the most important characteristic parameters for fillers is their activity, which depends on several factors: surface development, i.e., the general contact surface of the filler particles with the polymer, energy factor associated with the increase of energy necessary to destroy the sample, geometric factor and therefore the shape of grains, structure, porosity, agglomeration, and the ability to form adhesive bonds at the polymer-filler interface [5,6].

The awareness of environmental protection evolving along with technological progress means that composites reinforced with natural fibers, so-called biocomposites, play an increasingly important role in many branches of the economy [7,8,9]. Biocomposites from plant and wood-based fibers are used in a growing range of products, including aerospace, automotive, packaging, furniture, and construction and building materials [10,11,12,13]. The development of biocomposites is possible due to the satisfactory properties they exhibit and the advantages of natural fibers in relation to the more commonly used synthetic fibers [14,15]. Advantages of using biocomposites:

- low density, high strength and stiffness,
- fibers come from renewable sources,
- fiber production requires low energy expenditure, also assumes CO2 absorption and recycling of oxygen to the environment,
- production is cheaper than synthetic fibers,
- low health risk during fiber processing,
- low emission of toxic vapors during the combustion of the depleted composite,
- low risk of damage to the equipment during production,
- flexibility during processing [16,17,18,19].

The use of biocomposites is also associated with disadvantages: lower durability than composites with synthetic fibers, high moisture absorption and therefore increased swelling, lower strength compared to synthetic fibers, and lower processing temperature limiting the selection of polymer matrix. Such limitations, however, are a driving force for scientists in order to overcome them and thus obtain multifunctional materials. 

Despite the lower cellulose content, as in the case of wood and typical fiber plants, agricultural products such as wheat straw, rice straw, corn cobs, and maize stalks are equally valuable sources of fiber [20]. For many centuries, straw was considered as a by-product of cereal production, used especially for feeding and bedding livestock. Moreover, it is used as an additive for fertilizers and biofuels and in the production of heating material in the form of a pellet. However, all the aforementioned possibilities of straw management present drawbacks, and in spite of the huge production of straw, new alternative methods for its use are still needed. The use of these agricultural crop residues could open new markets for wheat straw and improve the rural agriculture-based economy [14]. 

The aim of the work was an in-depth characterization of the properties of natural rubber (NR) composites filled with cereal straw against the background of NR vulcanizates with the addition of conventional active and semi-active fillers in the form of chalk, carbon black, silica, and talc. So far, there are only a few scientific reports in the literature that emphasize the advantage of using this valuable bio-filler in elastomer technology [21,22,23]. None of them, however, relate to a constructive comparison of the obtained results with other well-known additives. As part of this work, a number of tests were carried out, including investigation of vulcanization kinetics and the rheometric properties of elastomer mixtures, the mechanical properties of vulcanizates before and after thermo-oxidative aging, crosslinking density, hardness, dynamic mechanical, damping, and the barrier properties of elastomeric composites based on natural rubber with the addition of selected fillers in different contents. This research article provides the opportunity for the design of composites with a high level of applicability, exhibiting multifunctional properties, and matching the latest trends in bio-inspired materials. In addition, the presented results describe the potential and comprehensive benefits of using natural, renewable filler in combination with natural rubber.

## 2. Materials and Methods 

### 2.1. Materials

Polymer: Natural rubber (NR) RSS I was provided by Torimex-Chemicals Ltd. Sp. z o.o (Konstatntynów Łódzki, Poland). Crosslinking agent: Rubber mixtures were vulcanized with sulfuric crosslinking system consisting of sulfur (Siarkopol, Poland); mercaptobenzothiazole (Sigma-Aldrich, Schnelldorf, Germany); micro-sized zinc oxide (Huta Będzin, Poland); and stearin (SA, Avantor Performance Materials, Gliwice, Poland). Fillers: Cereal straw (several different types of straw mixed together: rye, wheat, triticale and oat) was collected from local farms. Dried straw was crushed using a Pulverisette 5 classic line planetary ball mill (Fritsch, Idar-Oberstein, Germany), grinding time 2 h, speed 300 rpm; chalk–Socal 31 (Solvay GmbH, Hannover, Germany). Talc–Naintsch A-20 (Luzenac Group, Toulouse, France); carbon black–N774 carbon black (MAKROchem S.A, Lublin, Poland); silica – AEROSIL^®^ A380 (Evonik Degussa GmbH, Essen, Germany).

Composition of typical elastomer mixture: NR rubber (100 phr–parts per hundred rubber), sulfur (2 phr), mercaptobenzothiazole (2 phr), zinc oxide (5 phr), stearin (1 phr), and fillers (0 phr for reference sample or 10, 20, and 30 phr for other composites).

### 2.2. Methods

The oil absorption number (DBPA) of fillers was measured according to ASTM D2414 using Absorptometer C (Brabender, Duisburg, Germany). Process parameters: sample weight 20 g, titration rate of 4.0 mL/min. The oil used in this study was dibutyl phthalate DBP.

Elastomer mixtures, based on natural rubber and fillers, were prepared using an internal measuring mixer N50 (Brabender, Duisburg, Germany) and next milled with a crosslinking agent in a laboratory two-roll mill (Bridge, Rochdale, UK). Process parameters: roll dimensions: D = 140 mm, L = 300 mm; the rotational speed of the front roll: Vp = 16 min^−1^; the friction and the width of the gap between rollers: 1–1.2, 1.5–3 mm; the average temperature of the rolls: about 30 °C.

Rheometric properties and vulcanization time of compounds were tested using rheometer, model—MDR (Alpha Technologies, New York City, New York, USA), according to standard ISO 6502. Determination of minimum torque (M_L_), maximum torque (M_H_), torque increase (ΔM), scorch time (t_s2_), the time required for the torque to reach 90% of the maximum achievable torque (t_90_), which is used as an indicator of optimum time cure, were taken from a vulcanization curve. 

The mixtures were cured at 160 °C, at 15 MPa pressure for a curing time consistent with the vulcanization parameters. Steel molds placed between the shelves of an electrically heated hydraulic press were used for vulcanization. 

Dynamic mechanical analysis (DMA) was performed based on changes in the dynamic moduli as a function of the oscillation strain using a rotational rheometer with a plate‒plate system, by Ares G2 Rheometer (TA Instruments, New Castle, UK). The test parameters: stress: from 0.1% to 100%; sample deformation rate: 10 rad/s; test force: 5 N; temperature: 25 °C.

The morphology of composites and degree of dispersion of the filler in the elastomer matrix were elevated using a DisperTester 3000 microscope (MonTech, Buchen, Germany). The measurements of the composites containing 10 and 30 phr of filler were tested according to the standard procedures in ISO 11345: 2006. The instrument’s precision telecentric optical system uses the reflected light method for obtaining high-resolution reflective images from the sample surface.

SEM images of natural rubber composites were taken using an LEO1450 SEM microscope (Carl Zeiss AG, Oberkochen, Germany). Prior to the measurement, vulcanizates were broken down using liquid nitrogen; their fractures were coated with carbon and next examined.

The crosslink densities (υ_e_) of filler-reinforced rubber composites were determined using an equilibrium swelling test, performed in toluene solvent at room temperature, based on the Flory‒Rehner equation (Equations (1) and (2)) [24].

(1) $$\upsilon_{e}=\frac{\ln\left(1-V_{r}\right)+V_{r}+{µV}_{r}^{2}}{V_{0}\left(V_{r}^{\frac{1}{3}}-\frac{V_{r}}{2}\right)},$$

where: $\upsilon_{e}$—the crosslink density (mol/cm^3^); V_0_—the molecular volume of solvent (106.7 cm^3^/mol); µ—the Huggins parameter of the rubber-toluene systems is given by Equation (2):

μ = 0.478 + 0.404∙V_r._ (2)

V_r_ is the volume fraction of elastomer in the swollen gel (Equation (3)).

(3) $$V_{r}=\frac{1}{1+Q_{w}\frac{\rho_{r}}{\rho_{s}}},$$

where: Q_w_—equilibrium swelling reduced by the filler content (x [phr])–Q_w_ = (100 + x/100); ρ_r_—density of rubber [g/cm^3^]; ρ_s_—density of solvent [g/cm^3^].

The tensile test was performed using a universal strength machine (Zwick, Ulm, Germany) equipped with an extensometer. The measurements were carried out at a crosshead speed of 500 mm/min in accordance with standard ISO 37 by using standard dumbbell-shaped samples. The average value of tensile, elongation, and modulus was recorded for 5 samples of composites. 

The tear test was performed as per ISO 34 for 3 samples of each composite using the Zwick testing machine (Zwick, Ulm, Germany) with the test speed 50 mm/min. The specimen was cut to 100 mm × 15 mm × 1mm “trousers” shape with a pre-cut of 40 mm at the centre.

The hardness was measured using a Shore A type durometer (Zwick, Ulm, Germany) and followed the ISO 868 standard. The specimens were measured at 10 different locations on the composites. The average value of Shore A hardness number was calculated.

The thermo-oxidative degradation of the composites was performed at a temperature of 70 °C for 14 days, according to the PN-82/C-04216 standard. To estimate the resistance of the vulcanizates to aging, their mechanical properties after aging were tested and compared with the result obtained for samples before the aging process. The aging factor (K) was calculated as the numerical change in the mechanical properties of the samples upon aging (Equation (4)) [25]:

K = (TS ⋅ Eb)after aging /(TS ⋅ Eb)before aging,(4)

where: TS is the tensile strength of the sample and EB is the elongation at break.

The barrier properties were determined by the gas transmission rate (GTR) and coefficient of gas permeability (P), which were calculated from the following equations:

(5) $$GTR=\frac{V_{c}}{R\cdot T\cdot P_{u}\cdot A}\cdot \frac{\mathrm{dp}}{\mathrm{dt}},$$

(6) P=GTR·d

where: d—the thickness of the sample [m]; Vc—volume of low-pressure chamber [L]; T—temperature [K]; Pu—the gas pressure in the high-pressure chamber [Pa]; A—area permeation of gas through the sample [m^2^]; dp/dt—pressure changes per unit time [Pa/s]; R—gas constant 8.31·10^3^ [(L·Pa)/(K·mol)]

In the case of crosslinking density tests and mechanical properties, i.e., tensile strength, tear strength, and hardness, a statistical analysis based on standard deviation was calculated. 

## 3. Results and Discussion

### 3.1. The Oil Absorption Number (DBPA)

Measurement of the absorbance of dibutyl phthalate through the filler is an indirect measure of its activity providing information on the structure and porosity of the tested additive. The highest value of absorbed dibutyl phthalate was noted for silica (Table 1). The obtained result indicated that the filler was characterized by the most developed surface and the highest porosity and volume of free spaces between the aggregates of particles. This meant that the silica particles would probably show an enhanced ability to form their own “network” in the elastomeric medium. An excessively well-developed secondary filler structure in the polymer leads to the formation of agglomerates and aggregates, negatively affecting the properties of composites. The high DBPA value was also characterized by straw; its value (140 mL/100 g) was significantly higher than the other tested fillers. The structure is one of the factors affecting the so-called strengthening effect, characteristic for the group of active and semi-active fillers. Therefore, in the case of bio-composites filled with straw, improvement in strength properties could be expected.

### 3.2. Dynamic Mechanical Analysis (DMA)

The difference (ΔG′) between the elastic modulus at low strain (G′0) and high strain (G′∞) is a measurement of the Payne effect attributed to the filler structure and can be understood as physical bonds of the filler interaggregates (van der Waals, London forces) that are broken at high strains [26]. According to the theory of the Payne effect, low deformations are related to rubber-filler interactions and the high deformation to the filler‒filler ones [27]. The greater the ΔG′, the greater the Payne effect, indicating a greater breakdown of filler‒filler interaction, and the greater the amount of clusters present in the elastomeric matrix [28]. The Payne effect measurements were conducted up to 40–100% of strain. Unfortunately, it was not high enough to see the destruction of the secondary structure formed by the filler. However, it is possible to see the beginning of the destruction.

**Figure 1:**
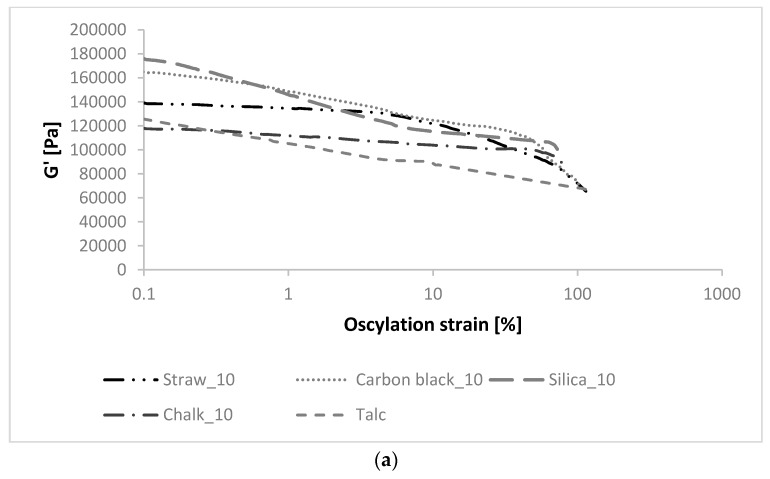

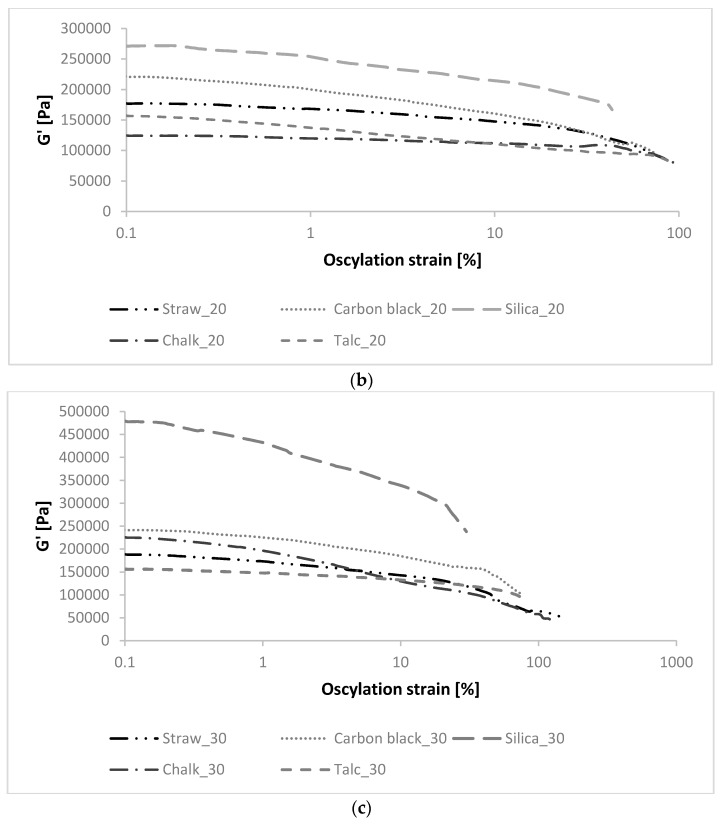
The storage modulus of composites containing (**a**) 10 phr, (**b**) 20 phr, and (**c**) 30 phr fillers as a function of oscillation strain.

The vulcanizates filled with silica and carbon black showed a larger G′ modulus at small strain, and the beginning of deformation of the filler network started earlier than the other fillers (Figure 1). In the case of straw, the course of the storage module curve was gentle, while the beginning of the deformation was observed at higher strain values, similarly to chalk and talc. During the destruction of the filler structure, the rubber associated with the elastomer matrix is released, which reduces its effective volume. This phenomenon is depicted in the form of a decrease in the modulus of elasticity. The lowest decrease in G′ values as a function of strain was observed in composites containing chalk and talc. The course of the storage module curve for composites filled with straw was similar to the vulcanizates with the addition of carbon black, which is considered a typical semi-reinforcing filler. Therefore, improvement of functional properties in composites made with the use of cereal straw is expected. However, the excessively high Payne effect (occurring for silica composites at higher contents) could be associated with a high tendency of the filler to agglomerate and aggregate in the polymer matrix, which negatively affects the properties of the composite [29]. The more agglomerated the filler, the higher the amount of rubber occluded into the aggregates, resulting in a greater hydrodynamic effect, which reduces filler-matrix interactions.

### 3.3. The Degree of Dispersion of the Filler in The Elastomer Matrix

The properties of the filler particles and the degree of filling in the polymer determine the intensity of interfacial interactions and the morphology of the system, which in turn affect the functional properties of the composite. As a result of binding with physicochemical forces, the primary particles of the filler form aggregate and agglomerate. The filler structure is understood as its own spatial network in the elastomer matrix. It depends on the content of the filler and its activity.

**Figure 2:**
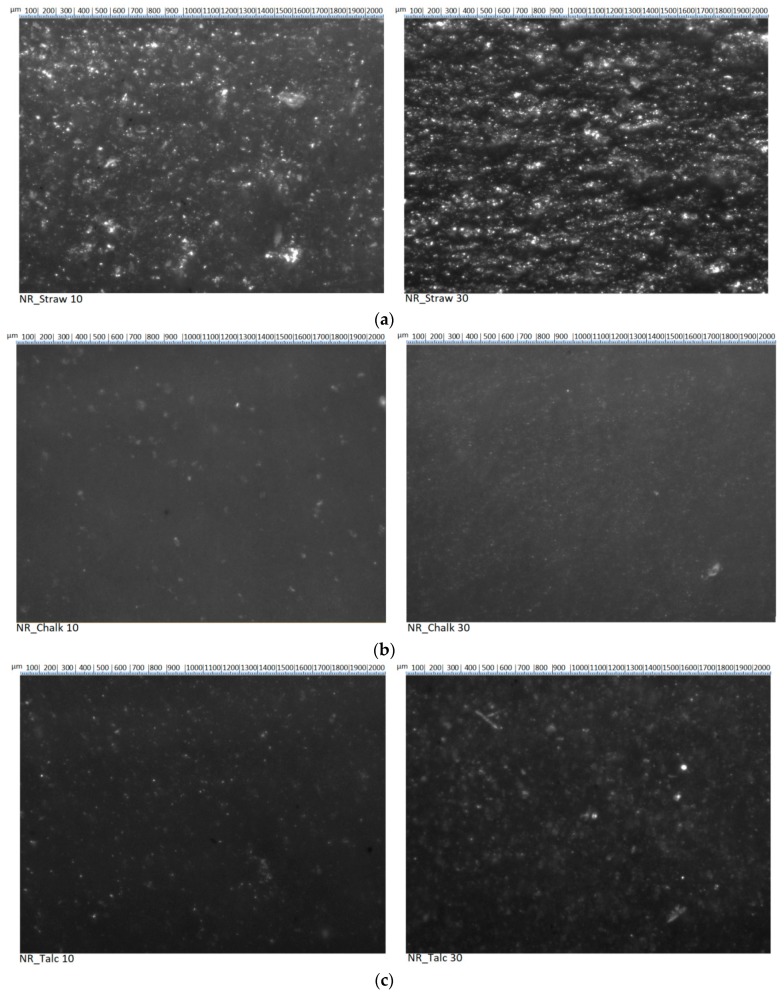

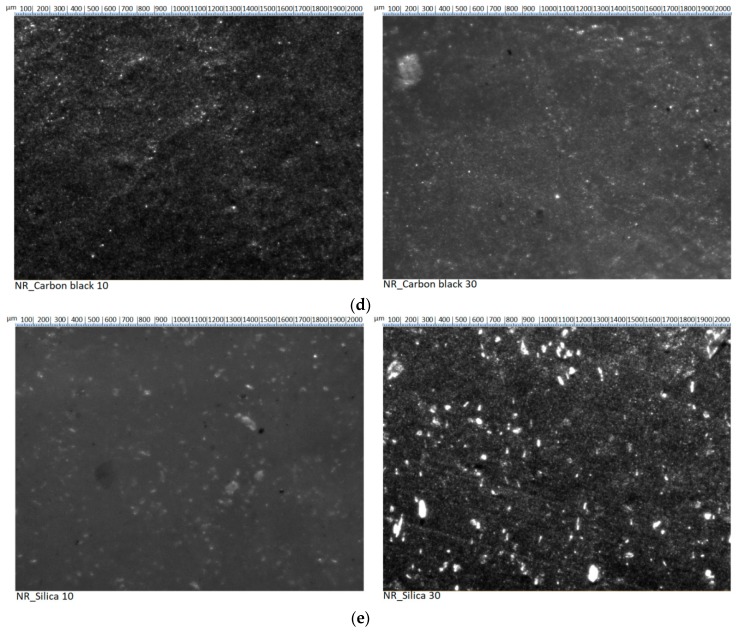
Images of composites and dispersion of the (**a**) straw, (**b**) chalk, (**c**) talc, (**d**) carbon black, and (**e**) silica in the natural rubber matrix.

The straw particles in the elastomer matrix exhibited a uniform distribution in the composite (Figure 2). However, their size was much larger compared to other fillers. Based on observations of images, it could be seen that the straw particles were different in size and shape but did not form agglomerates or aggregates. Increasing the content of lignocellulosic material to 30 phr did not adversely affect the distribution of particles in natural rubber. Definitely, the largest tendency for agglomeration was observed in the case of the application of nanosilica. Obtaining the appropriate degree of dispersion of the filler in the elastomer matrix was necessary both from the point of view of the macroscopic homogeneity of the material and the effectiveness of its reinforcing effect. Filler dispersion was a critical factor in determining the properties of filled rubber composites. Silica has a high density of silanol groups on the surface, which lead to strong filler–filler interactions and poor filler dispersions. The remaining fillers, similar to straw, were characterized by homogeneous distribution in the polymer matrix.

### 3.4. SEM Analysis

**Figure 3:**
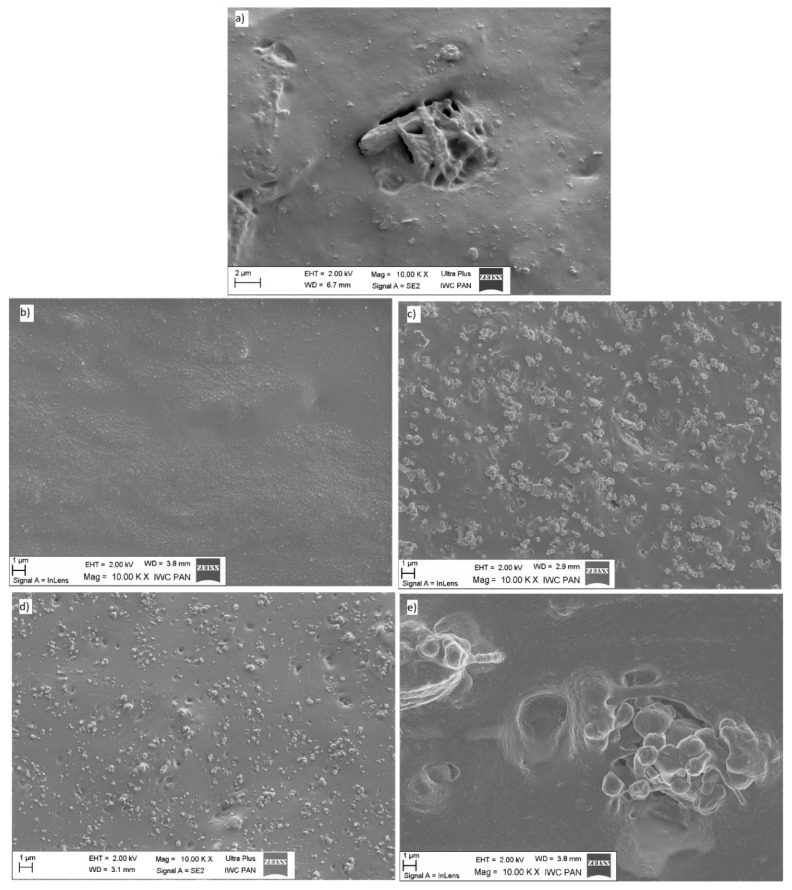
SEM images of composites filled with (**a**) straw, (**b**) chalk, (**c**) talc, (**d**) carbon black, and (**e**) silica.

In order to visualize the geometry of the filler particles and to present their degree of dispersion in the polymer, scanning electron microscopy (SEM) was performed. Analysis of SEM images (Figure 3) confirmed the results obtained by the light reflection method. Particles of fillers such as chalk, talc, or carbon black showed a high degree of dispersion in the elastomeric matrix. These fillers were uniformly distributed in the polymer. In addition, both particles and their clusters reached nanomometric sizes. On the other hand, nanometric silica exhibited a large tendency to accumulate into larger aggregates, forming agglomerates ranging from a few to a dozen micro-meters. In turn, the straw particles were characterized by a varied shape and size. Both nanometric particles and larger fibers with a size of a few micrometers were visible.

### 3.5. Rheometric Properties

**Figure 4:**
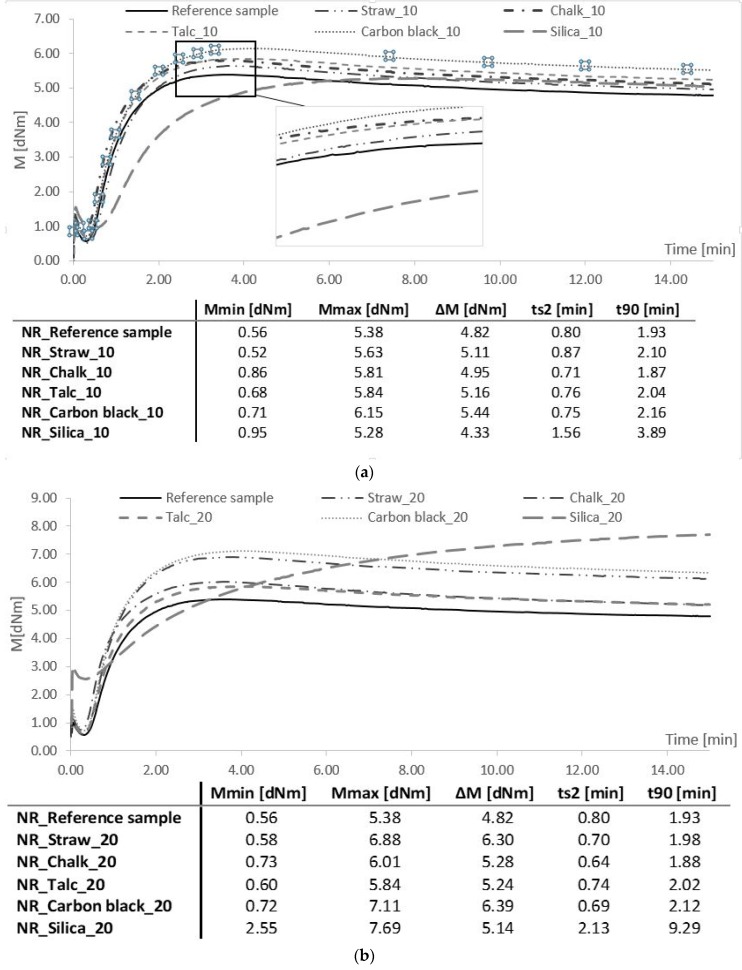

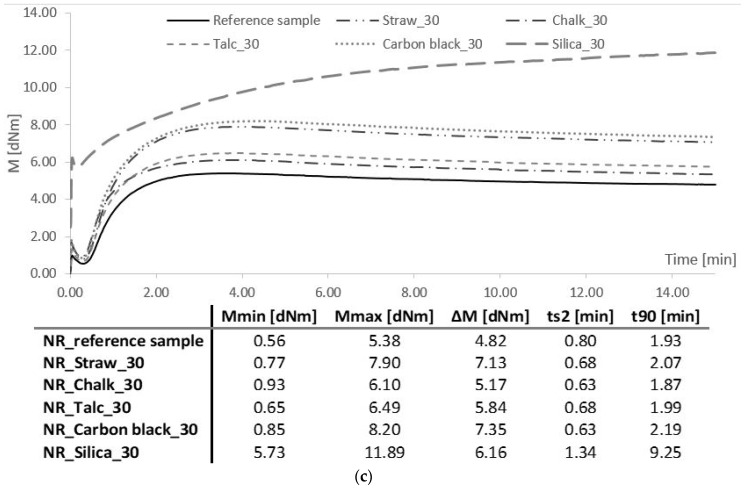
Rheometric properties and vulcanization parameters of elastomer mixtures filled with (**a**) 10 phr, (**b**) 20 phr, and (**c**) 30 phr.

The nature of the vulcanization process of mixtures containing straw was similar to composites filled with carbon black, talc, and chalk (Figure 4). These materials were characterized by a so-called plateau curve, while in the case of silica, the so-called curve with a marching module.

The smallest value of the minimum torque was shown by mixtures containing straw, which indicated that the biocomposites were characterized by the lowest viscosity among all tested materials. This is beneficial from a technological point of view. As the crosslinking reaction progressed, the stiffness of rubber mixtures filled with straw increased rapidly. The obtained maximum torque was close to the M_max_ value for the composites containing carbon black, indicating the high stiffness of crosslinked materials. Typically, a larger ΔM rheometric torque gain indicates an increased density of vulcanizates. The highest increase in torque gain during vulcanization was found for composites filled with carbon black and straw, which could be associated with an increased tendency for interactions between these fillers and natural rubber. The addition of straw to natural rubber, as well as talc, chalk, and carbon black, did not significantly affect the optimal curing time. All composites, except for those filled with silica, were characterized by a t_90_ value of about 2 min. Composites containing silica showed different rheometry properties compared to other vulcanizates, which could be related to the limited thermodynamic miscibility of the used filler with natural rubber. This was probably due to the formation of agglomerates in the composite, created with the increase in its content. Agglomerates hampered the crosslinking process, leading to a reduction in crosslinking density. In addition, the large surface area and activity of the silica contributed to the absorption of the interfering substances on its surface. This resulted in reduced efficiency in the vulcanization process. 

### 3.6. The Crosslinking Density

The properties of composites depend on the amount and type of filler and its characteristic features. The type of rubber, degree of crosslinking, and therefore the properties of the medium in which the filler is dispersed are equally important. 

The crosslinking density(ν_e_) of all filled vulcanizates was greater with respect to the reference sample (Table 2). Only composites filled with nanosilica showed a decrease in the υ_e_ value compared to the unfilled system. It was probably the effect of the poor activity of crosslinking agents in this system, which was confirmed by the previously presented rheometric curves. The highest crosslinking density was characterized by samples filled with carbon black and straw. Moreover, for the cited composites, along with the higher content of the filler, an increase in the υ_e_ value was observed. It is assumed that the construction of a spatial network of elastomers also depends on the interactions at the interface of the filler particle and the matrix. The strong filler‒polymer interactions behave like physical network nodes and act as additional elements in the composite network. Studies on the crosslinking density prove that straw, similarly to carbon black, can exhibit a semi-reinforcing effect.

### 3.7. Mechanical Properties

The conducted research showed that vulcanizates with straw, together with the increase in the filler content, show a smaller relative elongation (Eb) (Table 3). The composites were broken faster than the other samples, which proved their high stiffness. Samples filled with carbon black and silica presented similar relationships. The obtained results also indicated that the strength modules at 100 and 200% of elongation (SE_100_ and SE_200_) increased with the higher amount of straw, which proved the reinforcing effect of the lignocellulosic filler within the low elongation values. This phenomenon was not observed for composites containing chalk and talc.

**Figure 5:**
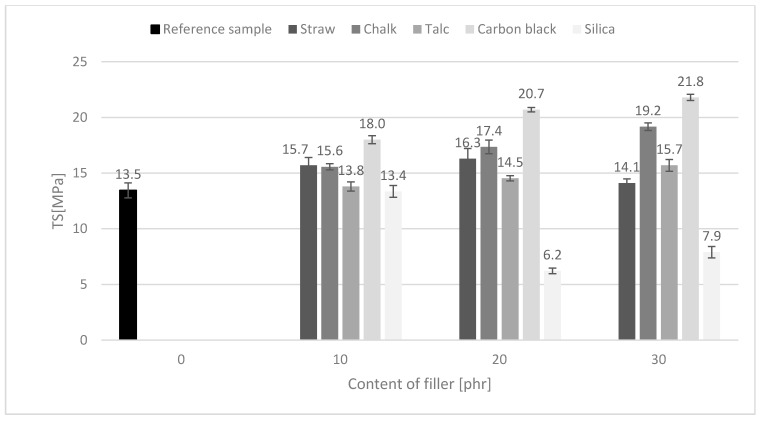
Influence of fillers on the tensile strength of the vulcanizates.

In addition, the tensile strength of composites containing 10 and 20 phr of straw oscillated at a comparable level to materials filled with commercially used chalk (Figure 5). It was higher than composites containing talc or nanosilica, which were already agglomerated at this content. However, the reduction of TS values for straw-based composites was observed at 30 phr. The decrease in the tensile strength and elongation of the vulcanizates could be caused by the fact that the short fiber with a high content influenced the increase of the shear forces during the stretching of the rubber macromolecules, thus reducing the mechanical strength of the vulcanizates [30]. Furthermore, the tensile strength of the polymers remains in close dependence on the adhesion between the filler and the medium as well as the mechanism of stress transfer in the filler-polymer system. As can be seen from the above-mentioned measurements of the crosslinking density, composites containing straw showed a significant degree of υ_e_ value. It is known that sites with higher crosslinking density and thus less mobility of polymer chains cause stress accumulation resulting in lower tensile strength.

### 3.8. Hardness

**Figure 6:**
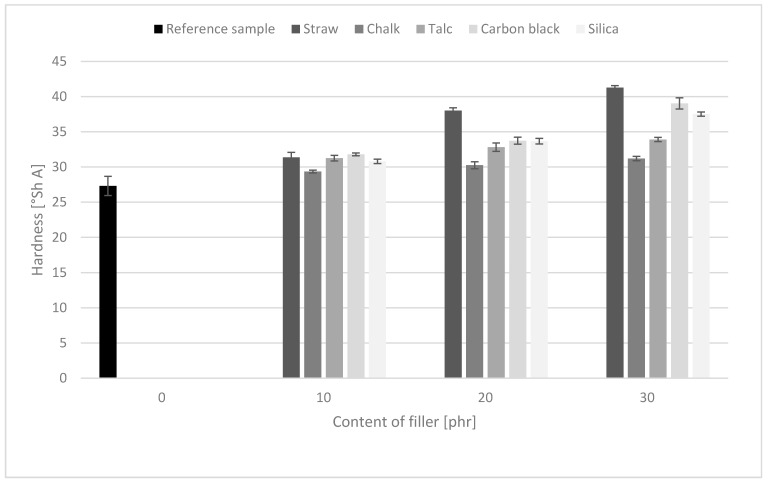
The hardness values of natural rubber vulcanizates containing different fillers.

The measurements of composite hardness values showed that composites containing lignocellulosic material were characterized by the highest hardness. The data presented in Figure 6 also shows that the hardness of the obtained composites increased with the addition of the filler. The obtained results confirmed that a larger increase in the ∆M torque gain values increased the vulcanizate hardness. Both results were a consequence of the stiffness of the material, resulting from the agglomeration of fillers in the elastomeric matrix and the crosslinking density, which directly influences the M_H_ value.

### 3.9. Tear Strength 

**Figure 7:**
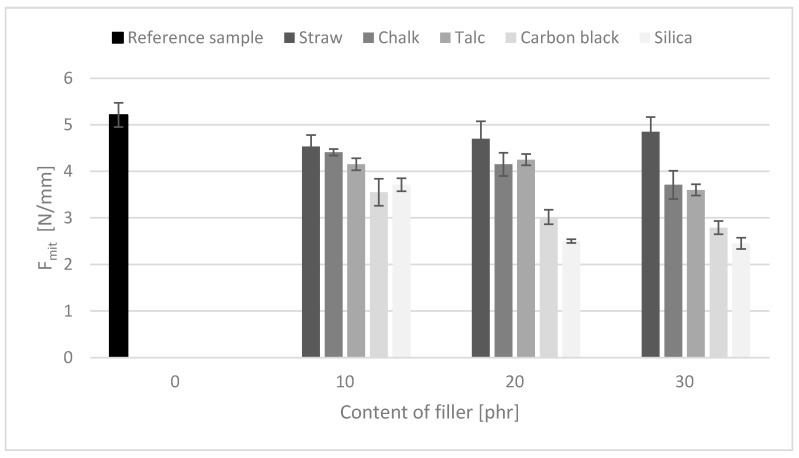
The tear strength of rubber composites.

All tested composites, regardless of the filler used, showed a decrease in tear resistance (Figure 7). The tear strength of composites ranged from 4.9 to 2.5 N/mm for NR_30 straw and NR_30 silica, respectively. The obtained values were lower than the strength of the reference sample (5.2 N/mm). Application of the lignocellulosic filler resulted in a reduction of the F_mit_ value to the smallest extent. The key aspect could be its morphology; the straw has a fibrous shape and the remaining fillers resemble spherical particles. The short fibers could be aligned along the direction of loading, which was perpendicular to the direction of tear propagation. Therefore, the short fibers transferred stress around and prevented crack growth. The concentration of straw increased tear strength by obstructing the tear path. A similar effect was observed by Kalaprasad [31]. 

### 3.10. The Thermo-Oxidative Degradation of the Composites

One of the research aspects discussed in this paper was the impact of the fillers used for natural rubber on changes in the physical and mechanical properties of composites during thermo-oxidative aging. The aging process led to a reduction in the tensile strength and elongation at break, which was probably the result of the increased density of vulcanizates. The unfilled natural rubber showed poor resistance to thermo-oxidative degradation processes (Table 4). The K coefficient (change of mechanical properties) after the aging simulation reached a value below one, which indicated significant negative changes in the energy of deformation. This was due to the chemical structure of the elastomer. Its structure contains double bonds, which are an active site for oxidation reactions, leading to degradation of the polymer. The addition of fillers affected the resistance of composites to aging in a diversified manner. The introduction of carbon black and talc resulted in a further small reduction in the aging factor. The silica-containing vulcanizates exhibited the highest value of the K coefficient, it was probably the effect of crosslinking of the material and not the action of the filler. These composites showed the marching nature of the rheometric curve. An increase in temperature during the aging simulation could lead to further crosslinking reactions and increased tensile strength of the composites. However, the use of straw as a filler of natural rubber improved its resistance to thermo-oxidation aging processes, which could have a positive effect on the exploitation values of such material. It is known that lignin’s hindered phenolic hydroxyl groups can act as a stabilizer of reactions induced by oxygen and its radical species [32]. Its reactivity with the radicals responsible for the oxidation is influenced by limited diffusion into polymers. 

### 3.11. Barrier Properties

The air permeability (P) of the composites was determined based on the rate of change in the pressure of the gas flowing through the sample. The fillers used had a significant influence on the barrier properties of the composites, in particular, due to their different morphology and geometry [33]. 

The GTR factor determines the rate of gas penetration through the sample. The addition of fillers contributed to the reduction of air permeability by the elastomer (Table 5). Composites containing silica and talc together with a higher content of fillers were characterized by increased gas permeability and faster diffusion through the sample. Agglomeration could lead to the formation of “interfacial voids” at the particle‒polymer interface and to the formation of a preferential pathway for the migration of the molecular penetrant [33,34]. In the case of the chalk addition, no significant changes in the GTR and permeability values were observed. Carbon black reinforced composites, on the other hand, reached the percolation threshold for the drop in air permeability at a 30 phr filler content. The value of gas permeation for samples containing straw decreased with the increase in the content of lignocellulosic material in natural rubber. Even at 20 phr of filler, the permeability value decreased significantly. The morphology of the filler used could be the key factor influencing the barrier properties of the composites. The introduction of the filler with fibrous morphology could result in the presence of separated layers of fiber in the polymer matrix, which was associated with the presence of a barrier effect and a reduction in the permeability of vapors and gases through the composite. Fiber imposes a more tortuous path for the noninteracting gas molecules to travel through the thickness of the composites [35].

## 4. Conclusions

The purpose of the work was to characterize cereal straw as a filler of natural rubber vulcanizates and to perform a comparative analysis with commonly used reinforcing and semi-reinforcing fillers used in elastomer technology. The literature data show that different types of natural fibers are usually compared between themselves or with synthetic fibers, min. i.e., glass, carbon or polymer, used as reinforcements in polymeric materials. In this case, the work is a review of selected processing, material, and utility properties of natural rubber biocomposites containing cereal straw, compared to conventionally produced composites filled with carbon black, silica, talc, and chalk. The conducted research proved that ground agricultural waste can successfully be used as a filler of elastomer mixtures.

The use of cheap and renewable raw material to strengthen the elastomer matrix contributed to the creation of an extensive secondary filler structure in the polymer, which was confirmed by dynamic mechanical analysis. Composites filled with lignocellulosic material were characterized by an optimal vulcanization time, similar to materials containing conventional fillers. In addition, straw-based biocomposites showed a large increase in the rheological moment, indicating their strongly developed spatial structure, which has been proven by studies of crosslinking density. Natural materials containing crop residues were characterized by a strong reinforcing effect at low strains of 100% and 200%. However, the high crosslinking density and the increase in stress concentration with a high content of short natural fibers led to reduction of elongation at break and tensile strength. Moreover, materials filled with straw in comparison with other composites were characterized by very good barrier properties. The fibrous straw particles form a structure that creates a gas permeable barrier, impeding the diffusion of air through the sample. All filled composites showed a higher hardness compared to the reference sample, which increased as the filler content was added. Vulcanizates filled with cereal straw were characterized by the highest hardness compared to other samples. Furthermore, as a lignocellulosic material, straw contains lignin in its composition, which is composed of compounds belonging to polyphenols that have antioxidant activity. Therefore, straw composites were characterized by higher resistance to thermo-oxidative aging compared to other tested materials.

In summary, the obtained results indicated that straw is an alternative source of elastomer matrix additive in comparison with typical fillers used in rubber technology, i.e., silica, soot, chalk, and talc. It should also be emphasized that its application in natural rubber is a new, alternative way of using plant-based waste such as straw. Its use provides the opportunity to design environmentally friendly materials containing natural and renewable resources, in line with the global trend of sustainable development and care for the environment. It is also an economically advantageous solution, allowing for the use of a cheap material, available worldwide, for which there is a production surplus and a development problem. 

## Figures and Tables

**Table 1:** Results of the oil absorption.

Filler	DBPA [mL/100 g]
Straw	139.6
Chalk	70.4
Talc	61.6
Silica	434.9
Carbon Black	72.6

**Table 2:** Effects of filler type and content on crosslinking density of the vulcanizates.

Filler	Content of Filler [phr]	ν_e_·10^−5^ [mol/dm^3^]
Reference sample	0	1.35 ± 0.04
Straw	10	1.84 ± 0.08
	20	2.39 ± 0.04
	30	2.76 ± 0.05
Chalk	10	1.97 ± 0.01
	20	1.85 ± 0.03
	30	1.91 ± 0.03
Talc	10	1.97 ± 0.02
	20	1.96 ± 0.02
	30	1.94 ± 0.03
Carbon black	10	2.17 ± 0.01
	20	2.44 ± 0.04
	30	2.64 ± 0.03
Silica	10	1.32 ± 0.01
	20	1.22 ± 0.03
	30	1.12 ± 0.02

**Table 3:** Mechanical properties composites filled with straw, chalk, talc, carbon black and silica.

Filler	Content of Filler [phr]	SE_100_ [MPa]	SE_200_ [MPa]	Eb [%]
Reference sample	0	0.69 ± 0.03	1.04 ± 0.01	691 ± 11
Straw	10	0.97 ± 0.01	1.43 ± 0.06	567 ± 18
	20	1.45 ± 0.05	3.16 ± 0.05	376 ± 15
	30	1.79 ± 0.06	4.58 ± 0.02	254 ± 11
Chalk	10	0.76 ± 0.02	1.14 ± 0.03	593 ± 21
	20	0.81 ± 0.03	1.24 ± 0.04	593 ± 22
	30	0.84 ± 0.04	1.32 ± 0.04	587 ± 18
Talc	10	0.80 ± 0.05	1.21 ± 0.04	594 ± 25
	20	0.87 ± 0.03	1.34 ± 0.06	592 ± 16
	30	0.96 ± 0.07	1.45 ± 0.03	597 ± 22
Carbon black	10	0.83 ± 0.01	1.30 ± 0.05	560 ± 17
	20	1.03 ± 0.03	3.75 ± 0.01	390 ± 14
	30	1.21 ± 0.05	3.25 ± 0.02	300 ± 14
Silica	10	0.77 ± 0.04	2.09 ± 0.03	586 ± 19
	20	0.81 ± 0.03	4.08 ± 0.03	412 ± 10
	30	1.19 ± 0.04	5.65 ± 0.05	384 ± 11

**Table 4:** Impact of different filler content on the thermo-oxidative aging of the composites.

Filler	Content of Filler [phr]	Before Aging		After Aging		K
		TS [MPa]	Eb [%]	TS [MPa]	Eb [%]	[-]
Reference sample	0	13.5	691	12.5	503.00	0.68
Straw	10	15.7	567	15.9	518	0.92
	20	16.3	376	15.2	358	0.89
	30	14.1	254	13.4	234	0.88
Chalk	10	15.6	593	14.7	446	0.71
	20	17.4	593	16.0	455	0.71
	30	19.2	587	14.0	459	0.57
Talc	10	13.8	594	12.9	436	0.69
	20	14.5	592	14.0	347	0.56
	30	15.7	597	15.0	369	0.59
Carbon black	10	18.0	560	16.4	391	0.64
	20	20.7	390	16.3	231	0.47
	30	21.8	300	14.6	215	0.48
Silica	10	13.4	586	12.8	419	0.69
	20	6.2	412	8.2	400	1.28
	30	7.9	384	9.7	312	0.99

**Table 5:** Gas transmission rate (GTR) and coefficient of gas permeability (P) of vulcanizates.

Filler	Content of Filler [phr]	P·10^−11^	GTR 10^−9^
		[mol/(m∙s∙Pa)]	[mol/(m^2^∙s∙Pa)]
**Reference sample**	0	1.53	13.58
Straw	10	1.16	11.44
	20	0.22	2.12
	30	0.17	1.42
Chalk	10	1.05	11.68
	20	1.01	10.89
	30	0.93	10.05
Talc	10	0.80	8.97
	20	1.06	10.22
	30	1.02	12.04
Carbon black	10	0.85	10.89
	20	0.82	10.15
	30	0.33	3.29
Silica	10	1.11	12.93
	20	1.43	13.73
	30	1.00	12.04
